# Effectiveness comparison of inpatient vs. outpatient pulmonary rehabilitation: a systematic review

**DOI:** 10.1186/s12913-022-08345-z

**Published:** 2022-08-12

**Authors:** Virginie Molinier, François Alexandre, Nelly Heraud

**Affiliations:** 1 Les Cliniques du Souffle – Groupe KORIAN, 800 Avenue Joseph Vallot, 34700, LODEVE, France; 2 Direction de la recherche et de l’innovation en santé - Groupe KORIAN, 800 Avenue Joseph Vallot, 34700, LODEVE, France

**Keywords:** Pulmonary rehabilitation settings, Effectiveness, Chronic respiratory disease, COPD

## Abstract

**Background:**

Pulmonary rehabilitation (PR) is the cornerstone of nonpharmacological treatments in chronic respiratory disease (CRD) management. PR can be performed in different settings, the most frequent of which are inpatient (inPR) and outpatient (outPR) management. In the literature, these two distinct modalities are generally considered to be the same intervention. Yet, they differ in terms of the length of stay, social support, and the time the patient is not in their normal environment, and the presumed absence of differences in terms of efficacy has never been established.

**Purpose:**

To identify studies that directly compared the effects of inPR and outPR on patients with all types of CRDs through a systematic review and to synthesize the evidence regarding the effectiveness comparison of both modalities.

**Methods:**

A literature search was performed on PubMed, Web of Science, and Cochrane Library on 24 March 2022. The inclusion criteria were: articles with adults with chronic respiratory disease and comparing inPR versus outPR in at least one PR outcome.

**Results:**

Seven hundred thirty-six articles were retrieved from the databases. Six retrospective articles met the inclusion criteria. A best-evidence synthesis (BES) was carried out. Eight outcomes could be found in the included papers. For healthcare burden and refusals, no data could be extracted, and thus no BES was performed. For the eight remaining outcomes, two results were in favor of inPR with moderate evidence (HRQoL and psychological status), three were in favor of no difference between inPR and outPR with moderate or limited evidence (muscle strength, dropouts/adherence, and survival status), and three led to conflicting results (exercise tolerance, dyspnea, and economic costs).

**Conclusion:**

With the current state of knowledge, the majority of the studies converge towards an absence of differences between inPR and outPR or in favor of inPR for seven out of eight outcomes, albeit with moderate, limited, or conflicting evidence. The greater effectiveness of inPR for some outcomes will have to be confirmed in a well-designed RCT in order to orient public health policies in terms of the development of PR with the best evidence-based medicine approach.

**Trial registration:**

PROSPERO: CRD42020166546.

**Supplementary Information:**

The online version contains supplementary material available at 10.1186/s12913-022-08345-z.

## Introduction

Pulmonary rehabilitation (PR) is the cornerstone of nonpharmacological treatment in chronic respiratory diseases to reduce the burden of the symptoms [1, 2]. Defined as a ‘comprehensive intervention based on a thorough patient assessment followed by patient-tailored therapies that include, but are not limited to, exercise training, education and behavior change’ [2], PR aims to 1) improve physical condition, exercise tolerance, health-related quality of life (QoL), and psychological condition, 2) reduce symptoms of dyspnea, and 3) favor long-term adherence to health-enhancing behaviors [2].

Due in particular to its multidisciplinary approach with coordinated health professional staff, PR was historically developed in a hospital context; either based on an outpatient setting (outPR) or a full-hospitalization setting (inpatient PR; inPR). Although other alternative models have also proven to be effective, such as community-based PR and home-based PR [3–5], to date, they remain marginal (provided in less than 5% of organizations [6]). There are no clear orientation criteria to favor one modality over the others in the PR statements [2, 7]. The choice to orientate a patient in one or the other modality hence appears to depend primarily on the available resources in each territory. Of the two hospital-based settings, outPR is the most common worldwide (provided in nearly 9 out of 10 organizations) [6]. However, inPR is the most common in some European countries (e.g., in France, 90% of stays are inPR - data from the French Technical Agency for Information in Hospitalization, 2018).

In the literature, these two distinct modalities are generally considered to be the same intervention. For example, in the Cochrane systematic review and meta-analysis of the effects of PR on health status, the two interventions were grouped together under the term “hospital-based programs” and the data were compiled jointly [7]. Furthermore, the setting is sometimes not disclosed in studies [8, 9]. Surprisingly, to our knowledge, no systematic review or meta-analysis based on the effects of PR has yet to address the equivalence between inPR and outPR.

Yet notable intrinsic elements indicate that inPR and outPR are likely to induce different effects. For example, in terms of program organization, inPR and outPR present different respective durations of programs and frequencies of sessions. While the majority of outPR programs last between 8 and 12 weeks, with 2 to 3 sessions per week [7], inPR programs are generally shorter (from 4 to 5 weeks) and the sessions are, therefore, spaced closer together (generally every weekday). The frequency of training sessions is an important parameter that can modulate the adaptations induced by exercise. For instance, at the same workload (i.e., the same intensity and the same total amount of exercise over the entire program), a greater frequency of resistance training (3 vs. 1 session per week) may result in greater improvement [10]. Another important intrinsic difference between inPR and outPR lies in the support model, whereby inPR requires that the patient is physically present 24/7 in the hospital. Notably, while inPR generates a definite break in patient routines for several weeks, outPR maintains patients in their usual environment. In addition, by its specific setting, inPR also offers more social support (e.g., informal caregiver support, other patient support, and health-caregivers present at all times). Yet, the level of social support has a key role in the efficacy of a therapeutic intervention [11]. More specifically, after a stay in outPR, it was shown that improvement in dyspnea was correlated with the level of social support [12]. Moreover, while it has been reported that, on average, one-third of patients do not complete their PR program in outPR (e.g., [13–16]), this issue is rarely described for inPR. This could be explained by the inPR environment that requires patients to be constantly present (day and night). In contrast, in outPR, the temptation not to return could be greater. This issue has, unfortunately, been investigated very little to date.

In the current state of knowledge, it remains unknown whether there is a loss of chance when one or the other modality is applied preferentially. Moreover, it is also unclear whether there is a risk of erroneous conclusions by analyzing outPR and inPR studies as being equivalent. In light of these considerations, we propose to perform a systematic review in order to identify studies that directly compared inPR versus outPR, as well as to synthesize the evidence regarding the effectiveness comparison of both modalities.

## Materials and methods

The protocol for this systematic review was developed according to the guidelines of the Preferred Reporting Items for Systematic Reviews and Meta-Analyses (PRISMA). The protocol has been registered in the international prospective register of systematic reviews PROSPERO (registration number CRD42020166546).

### Literature search strategy

The literature search strategies were developed using medical subject headings (MeSH) and text words. (Table 1) MEDLINE (PubMed platform), Web of Science, and Cochrane Central Register of Controlled Trials (CENTRAL) bibliographic databases were searched from database inception through March 24, 2022. The bibliographies of eligible articles as well as existing systematic reviews in the field were also screened.

**Table 1:** Search methodology for Systematic Review

Electronic database	Searching strategy
PubMed	(rehabilitation [Title/Abstract] OR rehabilitation [Text Word] OR readaptation [Title/Abstract] OR readaptation [Text Word]) AND (pulmonary [Title/Abstract] OR pulmonary [Text Word] OR respiratory [Title/Abstract] OR respiratory [Text Word]) AND (inpatient [Title/Abstract] OR in-patient [Title/Abstract] OR inpatient [Text Word] OR in-patient [Text Word]) AND (outpatient [Text Word] OR out-patient [Title/Abstract] OR outpatient [Title/Abstract] OR out-patient [Text Word])
Web of Science	(TS = readaptation OR TS = rehabilitation) AND (TS = pulmonary OR TS = respiratory) AND (TS = inpatient OR TS = in-patient) AND (TS = outpatient OR TS = out-patient)
COCHRANE	(rehabilitation OR readaptation) AND (pulmonary OR respiratory) AND (inpatient OR in-patient) AND (outpatient OR out-patient)

### Study selection

Articles were included if they met the following criteria: (1) the sample population consisted of adults (age > 18 years); (2) with chronic respiratory disease; (3) included in a pulmonary rehabilitation program (i.e., according to the international recommendations, PR must include exercise training and at least one of the following components: patient therapeutic education, breathing exercises, peer-group interaction, self-management skill development, or other recognized PR interventions along with optimization of pharmacotherapy), and (4) articles comparing inPR/outPR based on at least one of the PR outcomes. The selected studies were prospective and retrospective cohort studies and randomized trials that directly compared the two modalities: outpatient versus inpatient pulmonary rehabilitation. Book chapters, systematic reviews (exception for reference lists, which were checked as mentioned above), non-English articles, and conference abstracts without the full text were excluded. Two reviewers (F.A. and V.M.) screened the titles and the abstracts of the retrieved studies for relevance, and discrepancies were resolved by consensus. The reviewers were blinded to each other’s decision to include or exclude an article. Articles published in languages other than English were excluded after screening the title and abstract. Two reviewers (F.A. and V.M.) reviewed the remaining articles in their entirety for consistency with the study protocol. Discrepancies were resolved by a third reviewer (N.H.).

### Data extraction

The data extraction form for this systematic review was developed by the authors. The data collected included the following: (1) Type of study; (2) Study objectives; (3) Sample size; (4) Group assignment criteria, (5) Sample size per group, (6) Anthropometric characteristics, (7) Respiratory disease diagnosis, (8) Disease severity, (9) PR Program content, (10) Duration, (11) Number of sessions, (12) Intensity of exercise training, (13) Outcomes of interest found, and (14) Results from inPR versus outPR comparisons for each outcome.

### Risk of bias assessment

The risk of bias was studied by the modified Cochrane tool [17–20]. It included 13 types of biases: selection bias (criteria 1, 2, 9), performance bias (criteria 3, 4, 10, 11), attrition bias (criteria 6, 7), detection (or measurement) bias (criteria 5, 12), and reporting bias (criterion 8). The last criterion, “other” (criterion 13), was reserved for any type of potential bias that is not detected by the previous items. Two reviewers independently scored all the included studies according to the list of questions. They had to reach a consensus, otherwise, a third reviewer made the final decision. Low risk of bias was defined as 1) ‘*yes*’ having been answered to at least 10 questions and 2) with at least one ‘*yes*’ in each risk category. A moderate risk of bias was defined as 1) ‘*yes*’ having been answered to at least eight questions and 2) with at least one ‘*yes’* in two categories. All the other cases were considered to be ‘high risk of bias’.

### Best-evidence synthesis

Since a meta-analysis could not be performed due to the lack of homogeneity in the measured outcomes and a lack of data, a best-evidence synthesis (BES) [21, 22] was performed using the methodology from van Tulder et al. and Eijgenraam et al. [18, 23]. When reported, statistical values were included in our systematic review and the BES. The levels of evidence regarding the significance or non-significance of a relationship among studies were ranked according to the following statements:

1. ‘strong evidence’ was assigned if two or more studies with a low risk of bias and findings generally consistent in all studies (≥ 75% of the studies had consistent findings) reported a result;
2. ‘moderate evidence’ was assigned if a result has been reported by:
  One low risk of bias study and two or more moderate/high risk of bias studies.
  Or two or more moderate/ high risk of bias studies and consistent findings in all studies (≥ 75%);
3. ‘limited evidence’ was assigned if a result has been reported by:
  One or more moderate/high risk of bias studies or one low risk of bias study and consistent findings in all studies (≥ 75%);
4. ‘conflicting evidence’ was assigned in case of conflicting findings (< 75% of the studies reported consistent findings);
5. ‘no evidence’ was assigned when no studies could be found.

## Results

### Selection and search results

**Fig. 1:**
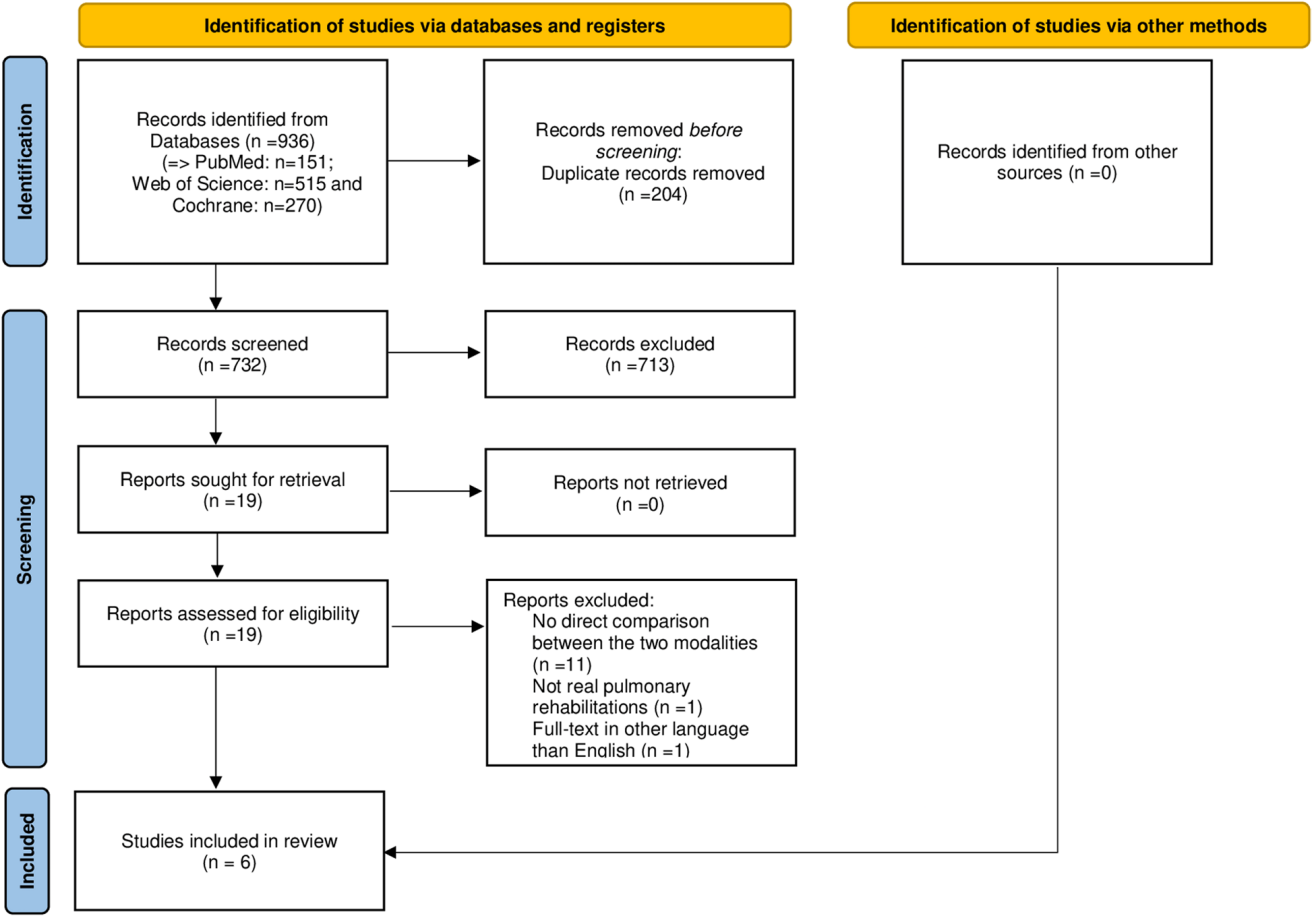
PRISMA Flow Diagram

After examining a total of 936 abstracts (732 after removal of duplicates), we retrieved 19 full-text publications for possible inclusion. Among these publications, we identified six studies comparing the effects of outPR versus inPR: Bowen et al. (2000) [24], Braeken et al. (2017) [25], Clini et al. (2001) [26] Guler et al. (2021) [27], Hjalmarsen et al. (2014) [28], and Stoffels et al. (2021) [29]. A flowchart describing the selection process is presented in Fig. 1. During the full-text article assessment for eligibility, the reasons for article exclusion were: absence of direct comparisons between modalities (*n* = 11), not a real PR program according to the official recommendations (*n* = 1), and articles not in English (*n* = 1). Among the included studies, several outcomes of interest were found [24–29]: health-related quality of life, exercise tolerance, muscle strength, psychological status, dyspnea, dropouts, economic costs, and survival rate.

## General description of the included studies

A general description of each study is presented in Table 2.

**Table 2:** Study characteristics and main results of the included studies

Author, Year, Country	Type of study	Total Sample size and per group	Group assignment criteria	Anthropometric characteristics	Respiratory disease diagnosis	Disease severity	PR Program content:	Duration (weeks), number of sessions, and Intensity of exercise training	Risk of bias	Outcome measure	Main results with mean average and [confidence interval] or (± standard deviation)
Bowen 2000 USA [24]	Retrospective study	*n* = 149 **inPR vs. outPR:** *n* = 36 vs. *n* = 113	n.a.	**inPR vs. outPR:** ***•*** age (years): 70 ± 6 vs. 69 ± 9 ***•*** BMI: 22.7 ± 6.3 vs. 25.5 ± 5.4 ***•*** Male patients: 33% vs. 49%	**inPR vs. outPR:** ***• ***n.a. vs. n.a. ***(total:****COPD (n = 133), chronic asthma (n = 12), chest-wall disease (n = 3), pulmonary fibrosis (n = 1))*	**inPR vs. outPR:** ***•*** FEV1 (% predicted): 30 ± 13 vs. 39 ± 19 ***•*** Dyspnea: n.a. ***•*** Number and type of comorbidities: n.a. ***• ***Exercise tolerance in baseline (6MWD in m): 422 ± 189 vs. 1123 ± 457	n.a.	***•*** n.a. ***•*** n.a. ***•*** n.a.	High	Survival status	No significant differences between inPR and outPR for survival status
Braeken 2017 Netherlands [25]	Retrospective study	*n* = 419 **inPR vs. outPR:** *n* = 261 vs. *n* = 158	Severity of the disease, co-morbid conditions, and access to nearby facilities (details not provided)	**inPR vs. outPR:** ***•*** age (years): n.a. vs. n.a. ***•*** BMI: n.a. vs. n.a. ***•*** Male patients: n.a. vs. n.a.	**inPR vs. outPR:** ***•*** COPD: *n* = 261 vs. *n* = 158	**inPR vs. outPR:** ***•*** FEV1 (% predicted): n.a. ***•*** Dyspnea: n.a. ***•*** Number and type of comorbidities: n.a. ***•*** Exercise tolerance in baseline (6MWD in m): 388 ± 120 vs. 502 ± 93	n.a.	***•*** inPR vs. outPR: 8 vs. 16 ***•*** inPR vs. outPR: 40 vs. 40 ***•*** n.a.	High	HRQoL	HRQoL improvement is significantly higher for inPR than for outPR for CAT, SGRQ-C, and CCQ (inPR: −3.8 [− 4.7–-2.9]/outPR: − 1.7 [− 2.7–-0.9] (*p* = 0.002); inPR: − 11.0 [− 12.7–-9.3]/outPR: − 6.1 [− 8.5–-3.7] (*p* = 0.001); inPR: − 0.8 [− 1.0–-0.7]/ outPR: − 0.2 [− 0.35–-0.1], (*p* < 0.001), respectively)
										Exercise tolerance	Exercise tolerance improvement is significantly higher for inPR than for outPR for 6MWD and no significant difference between inPR and outPR for CWRT (inPR: + 36.5 [27.5–45.5] m/outPR: + 0.7 [−7.3–8.7] m, (p < 0.001); inPR: + 196.4 s [158.6–234.1]/outPR: + 221.8 s [168.5–275.0] (ns), respectively)
										Psychological status	Psychological status improvement is significantly higher for inPR than for outPR for HADS anxiety and for HADS depression (inPR: −2.1 [− 2.6–-1.6] points/outPR: −1.1 [− 1.6–-0.6] points (*p* = 0.005); inPR: − 2.6 [− 3.1–-2.1] points/outPR: − 1.3 [− 1.9–-0.8] points (*p* < 0.001), respectively)
										Dropouts/adherence	No significant differences between inPR and outPR for dropouts/adherence
Clini 2001 Italy [26]	Retrospective study	*n* = 86 **inPR vs. outPR:** *n* = 43 vs. *n* = 43	Travel time from home (if > 1 h then inPR)	**inPR vs. outPR:** ***•*** age (years): 64 ± 8 vs. 64 ± 7 ***•*** BMI: 22 ± 4 vs. 24 ± 3 ***•*** Male patients: 67.4% vs. 67.4%	**inPR vs. outPR:** ***•*** COPD: *n* = 34 vs. *n* = 34 ***•*** Asthma *n* = 9 vs. *n* = 9	**inPR vs. outPR:** ***•*** FEV1 (% predicted): 57 ± 28 vs. 53 ± 21 ***•*** Dyspnea (Borg scale): 6.4 ± 1.6 vs. 8.5 ± 1.9 ***•*** Number and type of comorbidities: n.a. ***•*** Exercise tolerance in baseline (VO_2_ peak in mL kg min^− 1^): 15 ± 4 vs. 10 ± 8	***•*exercise training** (supervised incremental exercise until the patient achieved 30 min of continuous cycling at 70 to 80% of the maximal load achieved on an incremental cycle ergometer exercise test carried out at hospital admission) ***•*resistance training** (abdominal muscle activities, upper and lower limb muscle activities lifting weights progressively (from 300 to 500 g), and shoulder and full arm circling) ***•*education** (diagnosis-specific education sessions and nutritional programs and psychosocial counseling)	***•*** inPR vs. outPR: 3 vs. 8 ***•*** inPR vs. outPR: 12 vs. 24 ***•*** 70 to 80% of the maximal load	High	Exercise tolerance	No significant differences between inPR and outPR for exercise tolerance in peak workload (inPR: + 20%/outPR: + 16%, ns)
										Dyspnea	No significant differences between inPR and outPR dyspnea in Borg scale (inPR: −34%/outPR-26%, ns)
										Economic costs	The total per session was higher in inPR than outPR (246.9 euro vs. 171 euro, respectively) The total per program was lower in inPR than outPR (2715.9 euro vs. 3591 euro, respectively) The Grand total (including transports costs) was lower in inPR than outPR (2720 euro vs. 3677.7 euro, respectively)
Guler 2021 Canada, USA, Australia, Germany, and Switzerland [27]	Retrospective study	*n* = 701 **inPR vs. outPR:** *n* = 196 vs. *n* = 505	n.a.	**inPR vs. outPR:** ***•*** age (years): 70 ± 11 vs. 69 ± 12 ***•*** BMI: 26.8 ± 6.2 vs. 29.9 ± 6.2	**inPR vs. outPR:** fibrotic ILD: *n* = 196 vs. *n* = 505	**inPR vs. outPR:** ***•*** FEV1 (% predicted): 66 ± 20 vs. 72 ± 22 ***•*** Dyspnea: n.a. ***•*** Number and type of comorbidities: n.a. ***•*** Exercise tolerance in baseline (6MWD in m) 262 ± 128 vs. 358 ± 125	***•*exercise training** (aerobic training and/or interval training with a gradual symptom-limited increase in workload) ***•*resistance training: (**resistance training covered all large muscle groups and typically consisted of three sets with 8–12 repetitions) ***•*education** (educational sessions typically covered information on the pathophysiology of lung diseases, oxygen and medication use, symptom control, coping mechanisms, and self-management, with additional counseling on smoking cessation, nutrition and psychological support if needed)	***•*** inPR vs. outPR: 2–4 vs. 6–12 ***•*** inPR vs. outPR: 20–80 vs. 12–36 ***•*** gradual symptom-limited increase in workload.	High	Exercise tolerance	Exercise tolerance improvement is higher for inPR than for outPR for 6 MWD ((inPR: + 55 (±83) m/outPR: + 34 (±65) m
										Dropouts/adherence	No statistics were performed for this outcome
										Survival status	No statistics were performed for this outcome
Hjalmarsen 2014 Norway [28]	Retrospective study	*n* = 144 **inPR vs. outPR:** *n* = 72 vs. *n* = 72	n.a.	**inPR vs. outPR:** ***•*** age (years): 67.5 ± 8.2 vs. 70.4 ± 9.1 ***•*** BMI: 25.4 ± 5.6 vs.25.4 ± 5.6 ***•*** Male patients: 56.9% vs. 44.4%	**inPR vs. outPR:** ***•*** COPD: *n* = 72 vs. *n* = 72	**inPR vs. outPR:** ***•*** FEV1 (% predicted): 54.5 ± 21.8 vs. 52.2 ± 17.7 ***•*** Dyspnea: n.a. ***•*** Number and type of comorbidities: n.a. ***•*** Exercise tolerance in baseline (6MWD in m) 379.9 ± 128.8 vs. 367 ± 158	***•*exercise training and resistance training** (2 hours of exercise training daily, including endurance and strength training. Ventilatory muscle training was part of the exercise program. The endurance and strength training programs involved both upper- and lower extremity training on fitness center equipment including arm ergometer, cycle ergometer, and treadmill ***•*education** (12 lectures on patient education)	***•*** inPR vs. outPR: 4 vs. 8 ***•*** inPR vs. outPR: 20 vs. 16 ***•*** Mild and moderate COPD patients: High-intensity endurance training. Severe hypoxemic patients: low-intensity exercise keeping the SpO_2_ above 85% and the pulse below 130 per minute during activity	High	Survival status	No significant differences between inPR and outPR for survival status
Stoffels 2021 Netherlands [29]	Retrospective study	*n* = 625 **inPR vs. outPR:** *n* = 387 vs. *n* = 238	n.a.	**inPR vs. outPR:** ***•*** age (years): 66 ± 8 vs. 65 ± 8 ***•*** BMI: 25 [21–31] vs. 25 [22–30] Male patients: 45% vs. 59%	**inPR vs. outPR:** COPD: *n* = 387 vs. *n* = 238	**inPR vs. outPR:** ***•*** FEV1 (% predicted): 38 [28–54] vs. 51 [38–70] ***•*** Dyspnea (mMRC score): 3 [2–4] vs. 2 [2–3] ***•*** Number and type of comorbidities: n.a. ***•*** Exercise tolerance in baseline (6MWD in m): 332 ± 102 vs. 432 ± 88	***•*exercise training and resistance training:** (exercises to strengthen muscles of the upper and lower extremities, treadmill walking, stationary cycling, flexibility exercises, and daily supervised outdoor walks) ***•*education** (nutritional support, psychological counseling, and educational sessions	***•*** inPR vs. outPR: 8 vs. 14 ***•*** inPR vs. outPR: 40 vs. 40 ***•*** n.a.	High	HRQoL	HRQoL improvement is significantly higher for inPR than for outPR for CAT (**inPR** (*n* = 347): −3 (±6)/ **outPR** (*n* = 208): −1 (±6) (p < 0.001)
										Exercise tolerance	Exercise tolerance improvement is significantly higher for inPR than for outPR for 6MWD, CWRT TTE and 4MGS (**inPR** (*n* = 377): + 26 m (±59)/ **outPR** (*n* = 234): + 13 m (±47), (p = 0.002), **inPR** (*n* = 312): + 126 s [34–398] **outPR** (*n* = 227): + 77 s [−24–272] (*p* = 0.001), **inPR** (*n* = 387): + 0.1 m.s-1 [− 0.2–0.4]/**outPR** (*n* = 238): − 0.2 m.s^− 1^ [0.4–0], (*p* < 0.001), respectively) No significant difference between inPR and outPR for 5STS (**inPR** (*n* = 387) -1 s [− 4–1]/**outPR** (*n* = 238): − 1 s [− 4–0] (ns))
										Muscle strength	No significant difference between inPR and outPR for isokinetic quadriceps peak (**inPR** (*n* = 268): + 9 Nm [3–18]/**outPR** (*n* = 183): + 6 Nm [1–12] (ns))
										Dyspnea	Dyspnea improvement is significantly higher for inPR than for outPR for mMRC (**inPR** (*n* = 363): − 1 [− 2–0] points/**outPR** (*n* = 222): 0 [− 1–0] points (p < 0.001))
										Psychological status	Psychological status improvement is significantly higher for inPR than for outPR for HADS anxiety and for HADS depression (**inPR** (*n* = 345): −1 [−3–1] points/**outPR** (*n* = 208): − 1 [−2–1] points (*p* = 0.001), **inPR** (*n* = 345):-2 [−4–0] points/**outPR** (*n* = 208): − 1 [− 2–1] points (*p* < 0.001), respectively)
										Dropouts/ Adherence	No significant differences between inPR and outPR for dropouts/ adherence

***NOTE***: *PR* Pulmonary rehabilitation, *outPR* Outpatient PR, *inPR* Inpatient PR, *HRQoL* Health-related quality of life, *6MWD* 6-Minute Walk Distance, *CWRT* Constant Work Rate Test, *CWRT TTE* Constant Work Rate Test Time-to-exhaustion, *CAT* COPD Assessment Test, *SGRQ-C* St. Georges Respiratory Questionnaire for COPD patients, *CCQ* Clinical COPD Questionnaire, *HADS* Hospital Anxiety and Depression Scale, *5STS* Five Times Sit to Stand Test, *4MGS* Four-meter Gait Speed, *BMI* Body mass index, *VO*_*2*_*peak* Peak oxygen consumption at max load, *n.a* Not available, *ns* No statistical difference

### Study design

All six included studies were retrospective (Table 2). The number of patients included in each study varied between 86 and 701: *n* = 149 in Bowen et al., *n* = 419 in Braeken et al., *n* = 86 in Clini et al., *n* = 701 in Guler et al., *n* = 144 in Hjalmarsen et al., and *n* = 632 in Stoffels et al. All six studies included exhibited a high risk of bias. Details of the risk of bias are provided in Supplementary Data 1.

### Population characteristics

The population characteristics are listed in Table 2. The populations of three studies were exclusively composed of COPD patients [25, 28, 29]. In the other studies, the populations also included patients with asthma [30, 31], chest-wall disease [30], and pulmonary fibrosis [30, 32]. The baseline disease severity was reported in some studies using different indicators, such as the FEV_1_ [24, 26–29], dyspnea [26, 29], and exercise tolerance [24–29]. However, the number and type of comorbidities were never reported.

### Group assignment criteria

In the study by Braeken et al. [25], the two PR groups were formed according to disease severity, co-morbid conditions, and access to nearby facilities (but no details were provided). In the study by Clini et al. [26], the two PR groups were formed according to travel time from home (> 1 h inPR, or outPR otherwise). For the four other studies, the assignment criteria were not specified.

### Pulmonary rehabilitation characteristics

The program duration ranged from 2 to 16 weeks across the studies. The number of sessions also differed (12 to 80 sessions). In all six studies, the authors stated that the PR programs followed current guidelines, but the program details were sometimes lacking. Indeed, only four studies provided additional details about the PR program contents and intensities [26–28] (Table 2). In these studies, the intensity of the exercise training was similar between inPR and outPR [26–28].

## Comparison of outpatient and inpatient pulmonary rehabilitation programs and best-evidence synthesis assessment

Table 3 lists the best-evidence synthesis for each outcome. The best-evidence synthesis provided:

- moderate evidence *in favor of inPR* for health-related quality of life and psychological status
- moderate evidence *in favor of no difference between the two modalities* for dropouts/adherence and survival status
- limited evidence *in favor of no difference between the two modalities* for muscle strength
- conflicting evidence *in favor of inPR* or *in favor of no difference between the two modalities* for exercise tolerance and dyspnea,
- conflicting evidence *in favor of inPR* or *in favor of outPR* for economic costs
- no evidence for healthcare burden and refusals

**Table 3:** Best-evidence synthesis

Outcomes	Significant differences in favor of inPR: Study (Outcome) [Risk of Bias]	Significant differences in favor of outPR Study (Outcome) [Risk of Bias]	No difference between inPR and outPR Study (Outcome) [Risk of Bias]	Best-evidence synthesis
**Health-related quality of life**	Braeken et al. (CAT) [HR] Braeken et al. (SGRQ-C) [HR] Braeken et al. (CCQ) [HR] Stoffels et al. (CAT) [HR]	none	none	MODERATE EVIDENCE with four outcomes (100%) in favor of inPR
**Exercise tolerance**	Braeken et al. (6MWD) [HR] Guler et al. (6MWD) [HR] Stoffels et al. (6MWD) [HR] Stoffels et al. (CWRT TTE) [HR] Stoffels et al. (4MGS) [HR]	none	Braeken et al. (CWRT) [HR] Clini et al. (peak workload) [HR] Stoffels et al. (5STS) [HR]	CONFLICTING EVIDENCE with five outcomes (63%) in favor of inPR and three outcomes (37%) in favor of no difference between the two modalities
**Muscle Strength**	none	none	Stoffels et al. (isokinetic quadriceps peak) [HR]	LIMITED EVIDENCE with one outcome (100%) in favor of no difference between the two modalities
**Dyspnea**	Stoffels et al. (mMRC) [HR]	none	Clini et al. (Borg scale) [HR]	CONFLICTING EVIDENCE with one outcome (50%) in favor of inPR and one outcome (50%) in favor of no difference between the two modalities
**Psychological status**	Braeken et al. (HADS-anxiety) [HR] Braeken et al. (HADS-depression) [HR] Stoffels et al. (HADS-anxiety) [HR] Stoffels et al. (HADS-depression) [HR]	none	none	MODERATE EVIDENCE with four outcomes (100%) in favor of inPR
**Healthcare burden**	none	none	none	NO EVIDENCE
**Refusals**	none	none	none	NO EVIDENCE
**Dropouts/Adherence**	none	none	Braeken et al. [HR] Stoffels et al. [HR]	MODERATE EVIDENCE with two outcomes in favor of no difference between the two modalities
**Economic costs**	Clini et al. (total per program) [HR] Clini et al. (grand total) [HR]	Clini et al. (total per session) [HR]	none	CONFLICTING EVIDENCE with two outcomes (67%) in favor of inPR and one outcome (33%) in favor of outPR
**Survival status**	none	none	Bowen et al. [HR] Hjalmarsern et al. [HR]	MODERATE EVIDENCE with two outcomes (100%) in favor of no difference between the two modalities

***NOTE***: *PR* Pulmonary rehabilitation, *outPR* outpatient PR, *inPR* Inpatient PR, *HRQoL* Health-related quality of life, *6MWD* 6-MinuteWwalk Distance, *CWRT* Constant Work Rate Test, *CWRT TTE* Constant Work Rate Test Time-to-exhaustion, *CAT* COPD Assessment Test, *SGRQ-C* St. Georges Respiratory questionnaire for COPD patients, *CCQ* Clinical COPD Questionnaire, *HADS* Hospital Anxiety and Depression Scale, *5STS* Five Times Sit to Stand Test, *4MGS* Four-meter gait speed, *HR* High risk

### Health-related quality of life

Data on health-related quality of life (main outcome) were available in only two studies (Braeken et al. and Stoffels et al.) [25, 29]. Breaken et al. [25] assessed HRQoL across three different tools (CAT, SGRQ-C, and CCQ) versus only one in Stoffels et al. (CAT) [29]. In total, four results were available regarding the HRQoL.

In both studies, inPR resulted in a greater health-related quality improvement than outPR (Tables 2 and 3). Given that the two studies reported consistent findings with a high risk of bias, the best-evidence synthesis provided moderate evidence of results *in favor of inPR* for health-related quality of life improvement.

### Exercise tolerance

Data on exercise tolerance data were available in four studies (Braeken et al. Clini et al., Guler et al., and Stoffels et al.) [25–27, 29]. Braeken et al. [25] studied exercise tolerance with 6MWD and CWRT, Stoeffels et al. [29] with 6MWD, CWRT TTE, 4MGS, and 5STS, Clini et al. [26] with peak workload and Guler et al. [27] with 6MWD. In total, eight results were available regarding exercise tolerance.

For five results, inPR resulted in greater exercise tolerance improvement than outPR, whereas for three others, no difference was observed between inPR and outPR. (Tables 2 and 3). Given these results and the high risk of bias assessed for each study, the best-evidence synthesis provided conflicting evidence *in favor of inPR* or *in favor of no difference between the two modalitie*s.

### Muscle strength

Data on muscle strength was only available in the study by Stoffels et al. [29]. No significant difference in isokinetic quadriceps peak torque improvement was found between inPR and outPR (Tables 2 and 3). Therefore, limited evidence *in favor of no difference between the two modalities* was provided by the best-evidence synthesis.

### Dyspnea

Data on dyspnea were available in two studies (Clini et al. and Stoffels et al. [26, 29]). Clini et al. [26] found no significant difference in dyspnea improvement between inPR and outPR, whereas Stoffels et al. [29] showed greater dyspnea improvement in inPR compared to outPR (Tables 2 and 3). Given both studies presented a high risk of bias and each one reported different results, the best-evidence synthesis provided conflicting evidence for dyspnea due to significant results in favor of inPR and *in favor of no difference between the two modalities*.

### Psychological status

Data on psychological status were available in two studies, Braeken et al. and Stoffels et al. [25, 29]. Both studies used HADS, with one anxiety score and one depression score. In total, four results for psychological status were available. For both studies, inPR yielded greater improvement in anxiety and depression scores compared to outPR (Tables 2 and 3). Given that the two studies reported consistent results with a high risk of bias, [25, 29], the best-evidence synthesis provided moderate evidence *in favor of inPR* for psychological status improvement.

### Healthcare burden

No study from this systematic review provided healthcare burden data.

### Refusals

Refusal data were not available in the studies. Braeken et al. [33] provided the number of non-attendance (*n* = 32), but it was not possible to infer the cases of refusals.

### Dropouts/adherence

Dropouts/Adherence data were only available in three studies [25, 27, 29], but Guler et al. [27] did not provide any statistics in their article regarding this comparison. Braeken et al. [25] found that the PR setting had no significant impact on dropout rates, and Stoffels et al. [29] reported no significant differences in adherence between inPR and outPR (Tables 2 and 3). Given the high risk of bias reported for both studies and consistent results in both studies, moderate evidence *in favor of no difference between the two modalities* was obtained.

### Economic costs

Economic cost data were available in one study (Clini et al.) [26]. In this study, the authors calculated three different total costs (total per session, total per program, and the grand total including transport costs) (Tables 2 and 3). For the total per program and the grand total, inPR was cheaper than outPR, whereas for the total per session, the opposite was observed. Given these results and the high risk of bias assessed for each study, the best-evidence synthesis provided conflicting evidence results *in favor of inPR* or *in favor of outPR*.

### Survival status

Data on survival status were available in three studies [24, 27, 28], but Guler et al. [27] did not perform any statistical comparison on this outcome. Bowen et al. [24] and Hjalmarsen et al. [28] found that the PR setting had no impact on survival rates for COPD patients with a time period ranging from 1 to 10 years (Tables 2 and 3). Given the high risk of bias for both studies and consistent results observed, moderate evidence *in favor of no difference between the two modalities* was obtained for survival status.

## Discussion

We performed a systematic review in order to identify studies that directly compared inpatient pulmonary rehabilitation (inPR) versus outpatient pulmonary rehabilitation (outPR), as well as to synthesize the evidence on the effectiveness comparison of both modalities. Six retrospective studies were identified after the systematic review process, all of which had a high risk of bias. No meta-analysis was possible due to the lack of homogeneity in the reported outcomes. However, a general best-evidence synthesis was carried out. Of the ten targeted outcomes, eight could be found in the included papers. For health-care burden and refusals, no data could be extracted and, thus, no best-evidence synthesis (BES) was performed. For the eight remaining target outcomes, two results were in favor of inPR with moderate evidence (health-related quality of life - HRQoL and psychological status), three in favor of no difference between inPR and outPR with moderate or limited evidence (muscle strength, dropouts/adherence, and survival status), and three led to conflicting results (exercise tolerance, dyspnea, and economic costs).

The BES indicated moderate evidence in favor of greater effects of inPR on HRQoL and psychological status. Despite a consensus on these two outcomes, the level of evidence was only moderate due to numerous biases in the studies. Indeed, they were both retrospective, with no randomization and with heterogeneous groups, especially regarding disease severity. Unfortunately, the difference in disease severity was not taken into account in the analyses. Since severe patients usually exhibit more progress than the less severe patients, due to more scope for improvement [33], the higher severity in the inPR group represents a potential confounding factor, making it impossible to definitively conclude that inPR is superior to outPR for improving HRQoL and psychological status, despite a clear statistical trend.

For three other outcomes (muscle strength, survival status, and dropouts), the BES reported the absence of difference between inPR and outPR, with limited and moderated levels of evidence. While muscle strength is a key outcome in PR as an independent predictor of survival [34, 35], the fact that only one study investigated it is disappointing. Thus, the limited evidence on this result, in favor of an absence of a difference between the PR settings, does not allow any conclusions to be drawn. Regarding survival rates, the results revealed comparable survival rates with moderate evidence between both PR settings over a wide time frame after rehabilitation (up to 10 years) [24, 28]. No prospective follow-up was performed from the end of PR programs until the survival status was collected, translating to a real black box regarding any potential events that could have occurred during the follow-up. For example, neither of them reported some medium-term health indicators such as exacerbations and hospitalization rates (i.e., healthcare burden), which clearly influence survival status, however. As it stands, no modality can be considered more effective than the other in terms of the change in the vital prognosis of patients. A prospective study including several control variables appears to be essential to avoid any unwarranted conclusions regarding survival status. Regarding dropouts-adherence, the BES again showed no difference between inPR and outPR. To the best of our knowledge, in literature, the studies for which the main objective was to specifically investigate the dropouts phenomenon in PR were all carried out in outPR only [15, 30–32, 36, 37]. At first sight, due to the lack of studies in inPR, this may suggest that a high PR dropout rate is a phenomenon specific to outPR. Unexpectedly, our systematic review identified three studies that provided dropouts-adherence data in both PR settings, and with a significant number of dropouts in inPR in two studies [25, 29]. Unfortunately, they did not provide the reasons for dropping out. In previous studies performed in outPR, the main reasons for dropping out were mainly related to daily transport issues, lack of social support, and session times [15, 38]. By definition, these issues are unlikely in inPR. Beyond the rate of dropouts, the reasons for dropping out are of high importance in order to identify potential ways to minimize the dropout phenomenon. More specifically, if the reasons for dropping out differ between inPR and outPR, personalization of the PR setting according to each patient’s characteristics could become a way to limit dropouts in PR and thereby increase the PR efficacy. Hence, there is an urgent need for future studies to investigate and compare the reasons for dropping out between the two PR settings. Regarding the other three studies included in the review, the absence of data on dropout rates is problematic. Indeed, the efficacy of PR programs was only evaluated through the changes in the health status of patients who completed the program. However, the performance of a healthcare system must be assessed not only in terms of the health status of patients but also in terms of its capacity to be efficient for the majority of patients [39]. More specifically, if analyses were performed in per protocol (this information was not clearly provided in the three studies), failure to verify that completion or dropout rates were similar between inPR and outPR would be problematic. Indeed, in another study in which abandoners were followed and evaluated (intention to treat analyses), the results revealed differences in exercise tolerance according to the type of analyses: + 30 m in per protocol versus + 10 m in intention to treat analyses [39].

The BES also provided conflicting results between the two PR settings for exercise tolerance, dyspnea, and economic costs, preventing definition of any tendency. Concerning exercise tolerance, several different tests have been used to evaluate its progression. Some of them, such as the 6MWD, were in favor of a greater improvement in inPR. Interestingly, if only data on the 6MWD had been analyzed, moderate evidence would have been obtained. However, for some of the other variables, no difference between inPR and outPR was observed. Thus, the conflicting results could be explained by a lack of sensitivity of certain measures to detect any differences.

Concerning dyspnea, Stoffels et al. reported greater inPR efficacy, while Clini et al. did not find any differences between the two PR settings. Dyspnea is usually described as a multidimensional outcome, including impact, sensory, and emotional dimensions [40, 41]. In the two aforementioned studies, it was only assessed with unidimensional tools, and on two different dimensions: ‘impact’ in Stoffels et al. [29] vs. ‘sensory’ in Clini et al. [26]. Therefore, our systematic review could only provide a restrictive evaluation of dyspnea, and with conflicting results that could be explained by a difference in PR setting efficacy according to the dyspnea dimension. Finally, conflicting evidence in favor of inPR or in favor of outPR for economic costs was shown after BES. Only one study [26] investigated this outcome, and it was analyzed with different methods. First, the authors found that inPR in terms of the grand total (i.e., cost per program plus transports) was cheaper than outPR. This was expected since the inPR program included half the number of sessions than outPR (12 vs. 24 sessions, respectively). Given that no significant difference between inPR and outPR on PR outcomes was found in parallel, this result was logically interpreted as a possible indication of a better cost-benefit ratio for the inPR setting. However, it was also the case that the cost per session was cheaper in outPR than inPR, even without taking into account hospital bed costs for inPR. Consequently, it is likely that the inPR costs were underestimated. With different cost calculations yielding conflicting results it is, therefore, impossible to conclude regarding economic costs.

By drawing up a general summary, and despite the existence of biases and several different levels of evidence, it can be seen that the results of our comparison study are either in favor of inPR or in favor of the absence of differences between inPR and outPR. Not having a unanimous consensus regarding the superiority of one modality over the other raises questions about the impact of these results on the orientation of patients toward one or the other modality. Indeed, in light of the results, it would appear that the effectiveness comparison of inPR vs. outPR could be linked to the outcomes. Beyond a differentiated effect between the two modalities regarding some PR outcomes, it is also possible to envisage that inPR could be more suitable and adapted for certain patient profiles and outPR for some of the others. Unfortunately, this type of analysis at the individual level was not possible with the available data in the BES. Future research directions should also consider individual responses according to patient profiles, with the perspective of identifying the predictors of success across each PR setting.

### Methodological considerations of the systematic review

Best-evidence synthesis is a very relevant alternative that makes it possible to discern trends. It allows expression of results nuanced by different parameters such as the risk of bias of each included study, the number of studies, and the number of consistent results. Then, although this approach does not replace a meta-analysis, it allows extension of the knowledge with the available data. Several limitations in our systematic review warrant consideration. Best-evidence synthesis allows the level of interest found in the literature for a field to be highlighted. Here, we found a low number of studies. This clearly shows a lack of interest of the scientific community in this topic, which is nonetheless an essential question for patients. Then, when performing a best-evidence synthesis, a degree of subjectivity is introduced since the criteria of levels of evidence have to be defined even if it is based on a published methodology [18–23]. However, as explained by Slavin in 1986, in the absence of the possibility to perform a meta-analysis, best-evidence synthesis provides a means to combine the strengths of meta-analytic and traditional reviews [23]. A final weakness of our study is that we did not perform a literature search of unpublished papers or articles written in languages other than English.

## Conclusion

In conclusion, in the current state of knowledge, the majority of the studies converge towards the absence of differences between inPR and outPR or in favor of inPR for 7 out of 8 outcomes but with moderate, limited, or conflicting evidence. In addition, due to the retrospective nature of the studies, the absence of randomization, and of comparable severities between groups, no definite conclusions can be drawn from our systematic review and best-evidence synthesis. A well-designed RCT will potentially confirm this trend in favor of inPR in order to orient public health policies on the development of PR with a best-evidence-based medicine approach.

## Supplementary Information

**Additional file 1.**

## Data Availability

All data generated or analysed during this study are included in this published article and its supplementary information file.
