# Supplementary material for: Effectiveness comparison of inpatient vs. outpatient pulmonary rehabilitation: a systematic review
Source: BMC Health Serv Res. 2022 Aug 12;22:1028. doi: 10.1186/s12913-022-08345-z (PMC9373520; doi:10.1186/s12913-022-08345-z)
Supplement: Supplementary file 1 — Additional file 1. [file 12913_2022_8345_MOESM1_ESM.docx]

**Supplementary data**

**Table S1:** Risk of bias assessment for each study

|  | **SELECTION** | | | **PERFORMANCE** | | | | **DETECTION** | | **ATTRITION** | | **REPORTING** | **OTHER** | **RESULTS** |
| --- | --- | --- | --- | --- | --- | --- | --- | --- | --- | --- | --- | --- | --- | --- |
| **ARTICLE** | Selection (1) Was the method of randomization adequate? | Selection (2) Was the treatment allocation concealed? | Selection (9) Were the groups similar at baseline regarding the most important prognostic indicators? | Performance (3) Was the patient blinded to the intervention? | Performance (4) Was the care provider blinded to the intervention? | Performance (10) Were cointerventions avoided or similar? | Performance (11) Was the compliance acceptable in all groups? | Detection (5) Was the outcome assessor blinded to the intervention? | Detection (12) Was the timing of the outcome assessment similar in all groups? | Attrition (6) Was the drop-out rate described and acceptable? | Attrition (7) Were all randomized participants analyzed in the group to which they were allocated? | Reporting (8) Are reports of the study free of suggestion of selective outcome reporting? | Other (13) Are other sources of potential bias unlikely? |  |
| Bowen et al. | NO | NO | NO | NO | NO | UNSURE | UNSURE | NO | UNSURE | NO | YES | UNSURE | UNSURE | High Risk |
| Braeken et al. | NO | NO | NO | NO | NO | YES | UNSURE | NO | UNSURE | YES | YES | UNSURE | UNSURE | High Risk |
| Clini et al. | NO | NO | NO | NO | NO | YES | UNSURE | NO | NO | NO | YES | UNSURE | UNSURE | High Risk |
| Guler et al. | NO | NO | UNSURE | NO | NO | UNSURE | YES | NO | NO | UNSURE | YES | UNSURE | UNSURE | High Risk |
| Hjalmarsen et al. | NO | NO | YES | NO | NO | UNSURE | UNSURE | NO | UNSURE | NO | NO | UNSURE | UNSURE | High Risk |
| Stoffels et al. | NO | NO | NO | NO | NO | UNSURE | UNSURE | NO | NO | NO | UNSURE | UNSURE | UNSURE | High Risk |
